# Comparison between Conventional and Minimally Invasive Dynamic Hip Screws for Fixation of Intertrochanteric Fractures of the Femur

**DOI:** 10.1155/2013/484289

**Published:** 2013-08-26

**Authors:** A. Mahmood, M. Kalra, M. K. Patralekh

**Affiliations:** ^1^Department of Trauma and Orthopaedics, Milton Keynes Hospital NHS Foundation Trust, Milton Keynes, Buckinghamshire, MK6 5LD, UK; ^2^Department of Orthopaedics, Sohar Hospital, Sohar 321, Oman; ^3^Department of Orthopaedics, Dr. RML Hospital and Lady Hardinge Medical College and Associated Hospitals, Delhi 110001, India; ^4^Central Health Services, C1/160-161, Second Floor, Sector 16, Rohini, Delhi 110089, India

## Abstract

*Background*. Intertrochanteric fractures of the proximal femur are one of the most common fractures encountered, and dynamic hip screw with a side plate is the standard treatment. We compared a minimally invasive surgical technique with the conventional surgical technique used in the fixation of intertrochanteric fractures with the dynamic hip screw (DHS) device. *Methods*. Thirty patients with such fractures were treated with the conventional open technique and 30 with a new minimally invasive technique. Patients in both groups were followed up for 1 year. *Results*. There was less blood loss, minimal soft tissue destruction, shorter hospital stay, and early mobilization with the minimally invasive technique. *Conclusion*. The present study finds minimally invasive technique superior to conventional (open) DHS.

## 1. Introduction

Hip fractures are among the most common fractures encountered in orthopaedic trauma. Now they need more attention because as the average life expectancy, elderly population, and subsequent resulting osteoporosis continue to increase, orthopaedic surgeons will get more such cases [1–4]. Intertrochanteric fractures in the elderly are associated with high rates of mortality, ranging from 15 to 20%, as they are at a high risk for deep vein thrombosis (DVT), urinary tract infections, and pulmonary embolism if they fail to mobilize or ambulate early [5]. Surgical stabilization fulfills the aim of early mobilization and facilitates union in an anatomical position. Due to this, operative stabilization of these fractures is now the gold standard treatment. Although other options are available, the standard approach is to use a dynamic hip screw (DHS) with a 4-holed side plate in stable fractures in most centers [6–8].

DHS was historically introduced in 1950s to replace the standard fixed nail plate [4]. Traditionally a wide surgical exposure is necessary for this procedure which comes with its drawbacks like a large skin incision, considerable soft tissue trauma, significant blood loss, and pain. To avoid these problems minimally invasive surgery has been advocated recently [3, 4, 7, 9]. It has theoretical advantages of decreased blood loss, better cosmesis, less pain, and rapid rehabilitation. We conducted a prospective comparative study of conventional (open) DHS and minimally invasive DHS at our center to test the utility of this new approach.

## 2. Methods

This study was carried out in the Department of Orthopaedic Surgery, Sohar Hospital, Oman, between 2008 and 2011. A total of 60 patients with intertrochanteric femoral fractures were selected for this study and all of them gave informed written consent for the same. All the fractures were reduced and fixed with 135 degree dynamic hip screws with 4-hole side plate, 30 using conventional (open) technique (CDHS) and 30 with minimally invasive technique (MIDHS). Patients in both the groups were matched with respect to age, preoperative hemoglobin level, and morbidity. The same implants and instruments were used in both the groups. Preoperative and postoperative clinical details were recorded for all the cases. In particular, we also measured the difference between pre- and postoperative hemoglobin levels (hemoglobin drop), which is an indicator of blood loss. Patients received routine antibiotic prophylaxis given intravenously on induction of anaesthesia. The operating time was measured from beginning of skin incision to skin closure. In all cases of both conventional DHS group and minimally invasive group drains were removed 24–48 hours after surgery. All patients were rehabilitated using the same standard postoperative hip fracture management protocol by starting mobilization and weight bearing within 24 hours of surgery. The length of hospital stay was noted for each case and complications were also recorded for both the groups. The surgical technique for both the procedures is described below [3, 4, 7].

### 2.1. Conventional DHS

 A longitudinal skin incision 10–15 cm in length was made over the lateral aspect of upper thigh, starting from the middle of the greater trochanteric prominence and extending down the lateral aspect of femoral shaft. The fascia lata was incised longitudinally in the line of skin incision. The vastus lateralis muscle was split under direct vision. Fracture was reduced and its confirmation done with fluoroscope. Following fixation of the fracture in the standard fashion, a drain was used as per surgeon's preference, and the incision was closed in layers. 

### 2.2. Minimally Invasive DHS

**Figure 1:**
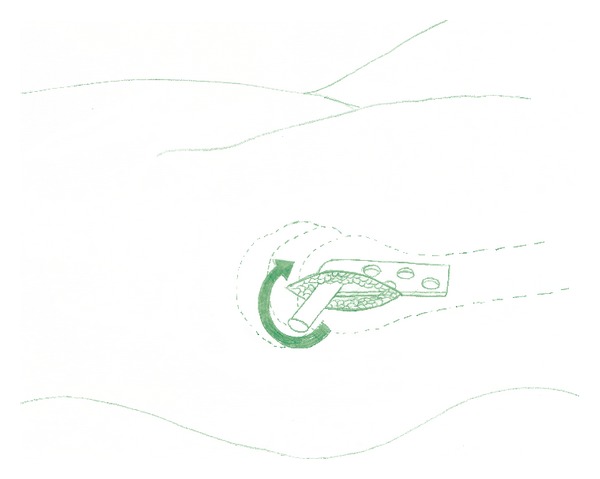
Technique of introducing DHS side plate in minimally invasive technique.

All fractures in this study received adequate closed reduction under c arm guidance (anatomical to 10° of valgus on anteroposterior radiograph and anatomical on lateral radiograph) prior to the start of operation. The incision was placed under fluoroscopic guidance by the identification of the site on the hip that corresponded to the position of the fracture. The size of the incision was not longer than 5 cm in any case. The iliotibial band and vastus muscles were split through one incision. After the insertion of a guide wire, reaming was carried out through this incision. The lag screw was inserted as usual and the guide wire was removed. After this barrel plate was also introduced through the same incision, turning the barrel from 180° to 90° as shown in Figure 1. The guide wire was then reintroduced through the side plate barrel and then rotated until the side plate lied suitably under the soft tissues. The guide wire was then passed through the lag screw under c arm guidance. The barrel was then engaged in the lag screw and advanced in the conventional fashion. The side plate screws were then placed in the usual manner through side plate holes by retracting the skin and subcutaneous tissue with a right angled soft tissue retractor. A drain was used according to surgeon's preference. The deep layers and the skin incision were closed in the usual fashion.

## 3. Results

Compared to patients in the conventional group, those in the minimally invasive group had shorter operating times (mean 50 versus 40 minutes) and a higher proportion of cases whose drain was removed within 24 hours (26% versus 71%). The drain output and decrease in haemoglobin level were not significantly different in the 2 groups. Patients undergoing minimally invasive DHS also had a shorter duration of hospital stay. There was no intraoperative and 30-day mortality in both the groups, but the 6-month mortality was 6.6% (2/30) in conventional group while being 0% in the minimally invasive group. While one-year mortality of 13.3% (4/30) was found in the conventional DHS group, 3.33% (1/30) was found in minimally invasive group. All patients achieved bone union within 4 months. Both groups have been compared in Table 1 and Figures 3, 4, 5, 6, 7, 8, 9, and 10.

## 4. Discussion

Hip fractures are a common cause of morbidity and mortality in the elderly population and are associated with considerable health expenditure. Although many internal fixation devices provide sufficient stabilization, the surgical treatment of intertrochanteric femoral fractures is still challenging [1]. The dynamic hip screw, which provides rigid fixation and allows early mobilization as it enables optimal collapse and compression of the fracture site [7], is the most common extramedullary device used for intertrochanteric fractures and has reasonable results [6, 8]. In addition, when compared with sliding hip screws, no definite evidence exists of a reduced failure rate with intramedullary nails in unstable intertrochanteric fractures [10]. Therefore, the routine use of intramedullary devices has not been recommended for the treatment of intertrochanteric fractures, and the dynamic hip screw is still the standard type of fixation for intertrochanteric fractures [6, 11].

However, insertion of this conventionally requires a 10–15 cm incision splitting the vastus lateralis, causing considerable bleeding and damage to the overlying soft tissues, and intertrochanteric fractures often occur in the elderly, who commonly have multiple comorbid conditions that may be worsened by the surgical trauma associated with a major operation [12]. In order to find less invasive techniques to simplify surgery and lower complication rate by reducing surgical time and blood loss, some authors used custom made implants or new devices (e.g., per cutaneous compression plate, PCCP) which require the purchase of additional instruments and implants by the hospital [3, 4, 7]. On the other hand, the minimally invasive DHS technique uses the existing instruments with which the operating team is familiar and confident, with no need to purchase new instruments. Several authors have shown that the same advantages can be gained by modifying the surgical approach while using existing fixation devices, thus requiring neither a new plating system nor training of operating theatre staff to familiarize with them. Therefore, the development of the minimally invasive dynamic hip screw technique, which causes less tissue damage and bleeding and shorter operative times and provides good fixation, may result in better outcomes, especially in elderly patients [13].

A reduced operative time, especially in elderly patients with comorbid conditions or poor cardiopulmonary reserve, is desirable because it reduces the risks of general anesthetic. This, along with reduced surgical trauma, may be significant in reducing postoperative morbidity and mortality in such patients. With regard to surgical blood loss, a previous angiographic study revealed that the average distance from the vastus lateralis ridge to the first significant perforating branch was 9.3 cm [3]. Therefore this area is relatively a safe vascular zone. In the minimally invasive dynamic hip screw technique, a 3–5 cm incision is made, and the incision point is approximately 4 cm below the vastus lateralis ridge. Therefore, blood loss decreases due to less soft tissue dissection and less fracture exposure and because an incision is made in the safe vascular zone [3, 4]. Because decreased blood loss is thought to be an explanation of reduced cardiovascular complications, which decrease the need for blood transfusion, this may have great clinical significance [9, 12]. 

**Figure 2:**
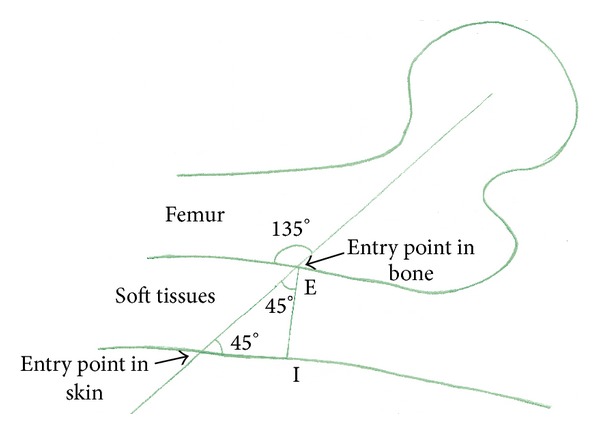
Guidewire entry point-isosceles right triangle technique.

**Figure 3:**
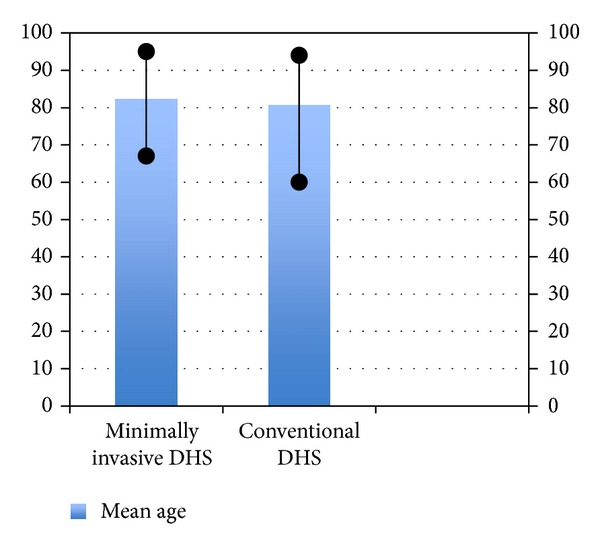
Bar diagram showing age distribution in the two groups.

**Figure 4:**
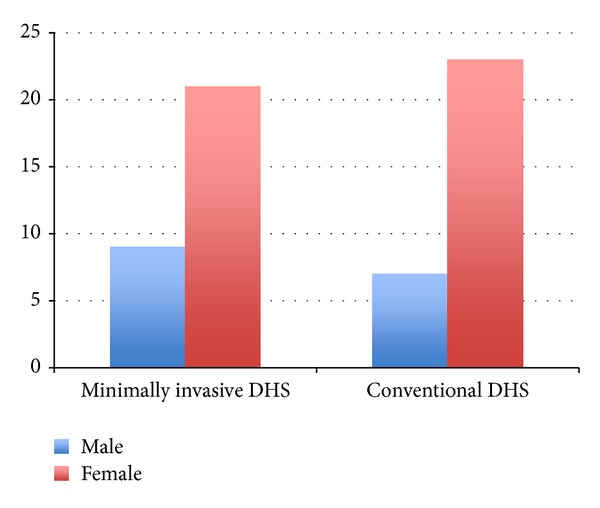
Bar diagram showing sex distribution in the two groups.

**Figure 5:**
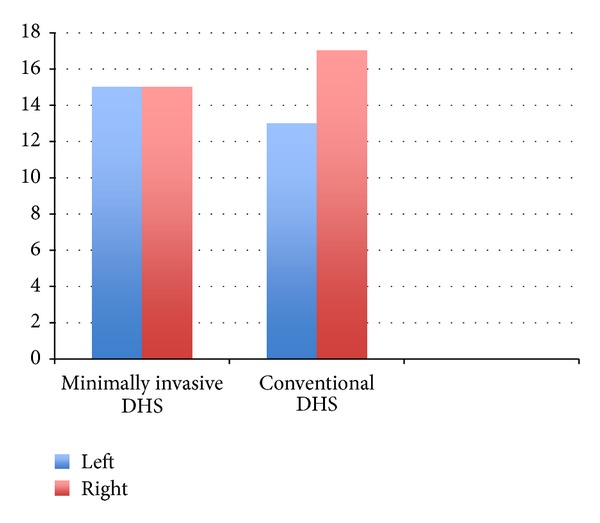
Bar diagram showing the side of fracture (left/right) in the two groups.

**Figure 6:**
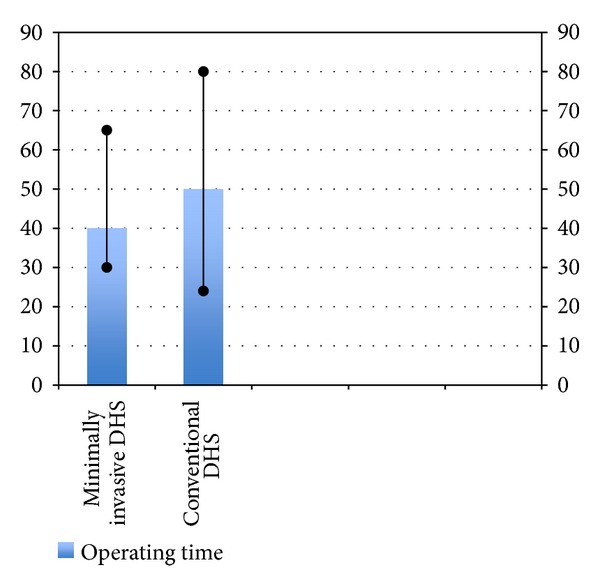
Bar diagram showing operating time in the two groups.

**Figure 7:**
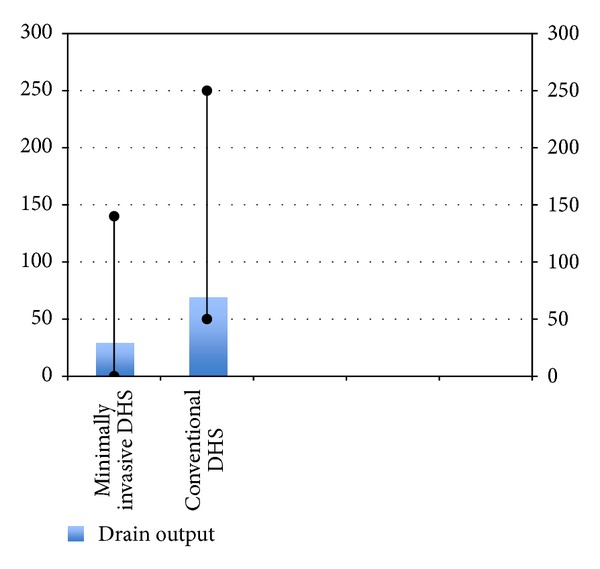
Bar diagram showing drain output in the two groups.

**Figure 8:**
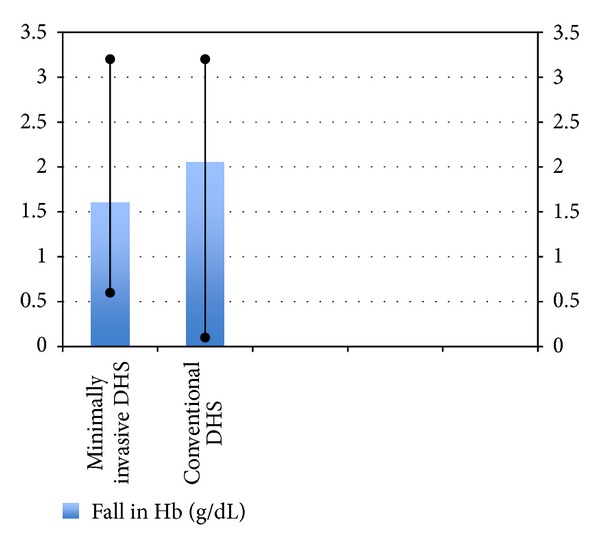
Bar diagram showing fall in Hb in the two groups.

**Figure 9:**
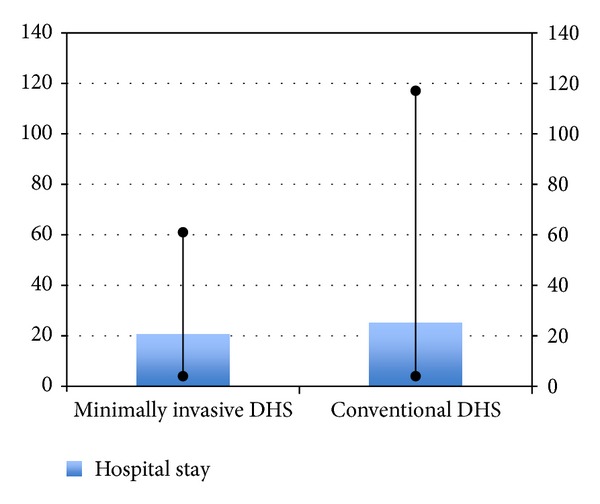
Bar diagram showing hospital stay in the two groups.

**Figure 10:**
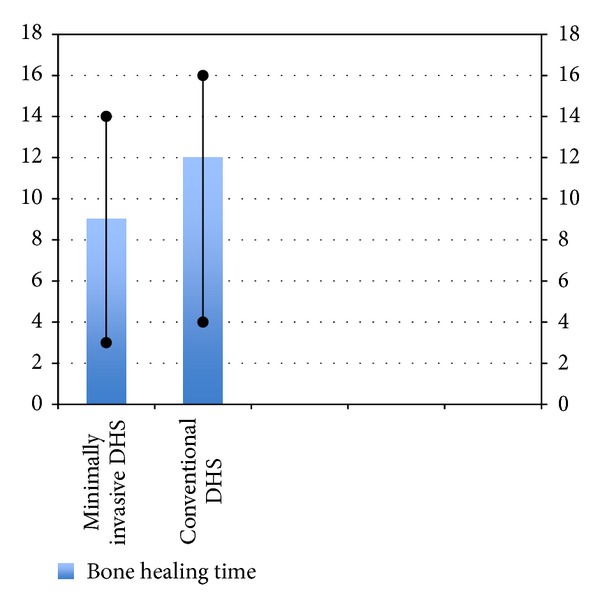
Bar diagram showing bone healing time in the two groups.

Wong et al. [4] proposed a novel and precise technique for determining the guide wire entry site which was the first step of their MIDHS surgical technique based on the concept of an isosceles right triangle (in such a triangle the two angles along the hypotenuse are equal to 45° and the external angle is 180° − 45° = 135° which is the same as the angle of the guide plate, as demonstrated in Figure 2. By placing a guide plate with guide wire on the anterior hip and placing the plate along the lateral border of the proximal femur, under fluoroscopic guidance, an AP line was traced along the femoral head and neck region along the guide wire and another along the side plate, with the two lines meeting at a point E. A lateral radiograph was then obtained, a guide wire was placed along the centre of femoral head and neck, and a lateral line was traced. A vertical line dropped from the previously mentioned point intersects this lateral line at another point I. Fluoroscopy is used to measure the distance of this point from skin to bone. This distance was then used to finally mark the entry site at the same distance along the lateral line distally. A 2.5 cm long incision was then made from the guide wire entry site distally.

Prete et al. [14] quantified the surgical trauma in conventional and minimally invasive surgical techniques for pertrochanteric fracture surgery based on measurement of markers of inflammation (interleukins). Significantly higher level of interleukin 6 was found in the postoperative period in patients operated by conventional technique than in those operated by minimally invasive technique. Results from the literature and their study indicate that IL-6 levels enable measurement of not only local tissue trauma/invasiveness but also the subsequent systemic response. By measuring the invasiveness of the intervention (second hit/additional tissue trauma) based on levels of interleukins, it could become possible not only to detect local damage but also to obtain an independent predictor of risk/outcome in elderly patients with pertrochanteric fracture. However a study by Lee et al. [7] showed a relatively high incidence of avascular necrosis of the femoral head with the minimally invasive technique. Vascular insult to the femoral head may therefore be considered as one potential drawback of this technique.

Zhou et al. [13] carried out a meta-analysis of studies on minimally invasive versus conventional DHS [3, 4, 7, 9] and found that there was a lower rate of serious postoperative complications in the minimally invasive dynamic hip screw group compared with the conventional dynamic hip screw group (relative risk, 0.35; 95% confidence interval (CI), 0.16, 0.78). Also the average operative time (weighted mean difference, −16.32; 95% CI, −28.78 to −3.86), hemoglobin decrease (weighted mean difference, −1.44; 95% CI, −1.98 to −0.89), and length of stay (weighted mean difference, −3.72; 95% CI, −5.44 to −2.01) were lower in the minimally invasive dynamic hip screw group, while the postoperative Harris Hip Score (weighted mean difference, 1.42; 95% CI, 0.23 to 2.60) was higher in the minimally invasive dynamic hip screw group.

Recent studies [15, 16] showed that stable pertrochanteric had successful fixation with two-hole DHS plate. It appears that the widespread use of four-hole side plate DHS in stable pertrochanteric fractures is merely based on tradition and needs to be reconsidered in the present era of evidence-based medicine. Biomechanical studies have also demonstrated equivalent peak load to failure when comparing the two- and four-hole DHS plates [17]. Therefore, using the two-hole DHS may be at least as safe as the four-hole DHS in nonosteoporotic patients, and the surgical exposure required will be smaller. It may also be economical, both financially and in the use of operating time. In osteoporotic bone, though, use of longer side plates may be safer [7].

Postoperatively, elderly patients are at a high risk for DVT, urinary tract infections, and pulmonary embolism if they fail to mobilize or ambulate early. Reduced operative time, less bleeding, and less postoperative pain may promote earlier ambulation in the minimally invasive dynamic hip screw group, which may contribute to a more favorable outcome in terms of serious postoperative complications [7, 13]. Also it may fasten overall patient recovery, thereby facilitating early discharge and, therefore, having significant financial implications for hospitals [13].

Because intertrochanteric fractures usually occur in elderly patients who may have cognitive deficits from age or have medical diseases, it is important for patients to return to preinjury activity levels as soon as possible to avoid complications. The minimally invasive dynamic hip screw, as it offers faster rehabilitation, is especially beneficial for older patients [4, 7, 13].

Our study was short term and had a relatively small number of patients. Further studies with larger number of patients, proper randomization, blinding procedure, and robust analysis will give more conclusive results.

To conclude, the minimally invasive dynamic hip screw and the conventional dynamic hip screw both are effective, simple, and safe for the treatment of intertrochanteric fractures. Compared with the conventional dynamic hip screw, the minimally invasive dynamic hip screw usually has a shorter operative time, less hemoglobin decrease, and a shorter length of stay, which benefit patients and reduce hospital costs and may therefore be recommended in place of conventional technique.

## Figures and Tables

**Table 1:** Demographic data, early and late postoperative parameters, and radiological findings.

Data	Conventional DHS	Minimally invasive DHS
Age (years)	80.7 (60–94)	82.2 (67–95)
Sex (male/female)	7/23	9/21
Side operated (left/right)	13/17	15/15
Operating time (minutes)	50 (24–80)	40 (30–65)
Drain output (mL)	70 (50–250)	30 (0–140)
Decrease in Hb level (g/L)	20.5 (1–32)	16 (6–32)
Duration of hospital stay (days)	25 (4–117)	20.5 (4–61)
Bone healing time (weeks)	12 (4–16)	9 (3–14)
